# Quantifying the Short-Term Costs of Conservation Interventions for Fishers at Lake Alaotra, Madagascar

**DOI:** 10.1371/journal.pone.0129440

**Published:** 2015-06-24

**Authors:** Andrea P. C. Wallace, E. J. Milner-Gulland, Julia P. G. Jones, Nils Bunnefeld, Richard Young, Emily Nicholson

**Affiliations:** 1 Department of Life Sciences, Silwood Park Campus, Imperial College London, Ascot, SL5 7PY, United Kingdom; 2 Centre for Environmental Policy, Imperial College London, Ascot, SL5 7PY, United Kingdom; 3 School of the Environment, Natural Resources and Geography, Bangor University, Bangor, LL57 2UW, United Kingdom; 4 Biological and Environmental Sciences, School of Natural Sciences, University of Stirling, Stirling, FK9 4LA, United Kingdom; 5 Durrell Wildlife Conservation Trust, Les Augres Manor, Trinity, Jersey, JE3 5BP, Channel Islands; 6 Department of Biology & Biochemistry, University of Bath, Bath, BA2 7AY, United Kingdom; 7 School of Life and Environmental Sciences, Centre for Integrative Ecology, Deakin University, Burwood, Victoria, 3125, Australia; Aberystwyth University, UNITED KINGDOM

## Abstract

Artisanal fisheries are a key source of food and income for millions of people, but if poorly managed, fishing can have declining returns as well as impacts on biodiversity. Management interventions such as spatial and temporal closures can improve fishery sustainability and reduce environmental degradation, but may carry substantial short-term costs for fishers. The Lake Alaotra wetland in Madagascar supports a commercially important artisanal fishery and provides habitat for a Critically Endangered primate and other endemic wildlife of conservation importance. Using detailed data from more than 1,600 fisher catches, we used linear mixed effects models to explore and quantify relationships between catch weight, effort, and spatial and temporal restrictions to identify drivers of fisher behaviour and quantify the potential effect of fishing restrictions on catch. We found that restricted area interventions and fishery closures would generate direct short-term costs through reduced catch and income, and these costs vary between groups of fishers using different gear. Our results show that conservation interventions can have uneven impacts on local people with different fishing strategies. This information can be used to formulate management strategies that minimise the adverse impacts of interventions, increase local support and compliance, and therefore maximise conservation effectiveness.

## Introduction

Natural resource management plans often fail to address the human dimensions of conservation [1–3]. Short-term costs to resource users are rarely quantified, and cost heterogeneities even less so, yet understanding and mitigating these costs is crucial for effective conservation planning to minimise impacts and lead to better compliance [4, 5]. Indeed, misunderstanding resource use and stakeholder behaviour may be one of the main causes of many management failures (see [6–8]).

Artisanal fisheries in developing countries are diverse and dynamic [9–11]. Spatial and temporal variations in resource availability, and in the types of gear used, are likely to lead to variation in catch. This diversity means that management interventions, such as no-take zones, seasonal closures, and the minimum length of fish that may be caught, are likely to have differential impacts on returns from fishing, and therefore livelihoods, for different groups of fishers [12]. Most artisanal fisheries are multi-species, multi-gear fisheries where traditional methods of stock evaluation are inappropriate [13]. However, catch and effort data from fisher reports, together with fishing location and gear characteristics as well as information regarding the time of year, can be used to understand resource (or catch) value to users.

The Lake Alaotra wetland is the primary inland fishery in Madagascar and a site of biodiversity conservation importance, providing the only habitat for the Critically Endangered Alaotran gentle lemur (*Hapalemur alaotrensis*). Current spatial and temporal conservation interventions for Lake Alaotra (described below) are being reviewed, making an investigation of the potential impacts of restrictions to various fishing groups and activities timely. This study aims to estimate the short-term costs which would be faced by fishers on first being subject to two management interventions; current restricted areas and a proposed earlier temporal closure (October-November). In particular, we examined how spatial and temporal patterns of total catch weight and fishing effort vary across the two primary methods used in the fishery, traps and gill nets, in order to evaluate the potential differential impacts of the two management interventions on the livelihoods of fishers using these methods. These transient costs are fundamentally important in order to understand fishers’ perceptions of, and subsequent response to, interventions. Post-adaptation costs are important in the longer term, but a focus on these can obscure the short-term hardship fishers may face, and their consequent resentment. The scope for rapid adaptation by fishers at Lake Alaotra may also be limited.

## Methods

### Study site

**Fig 1:**
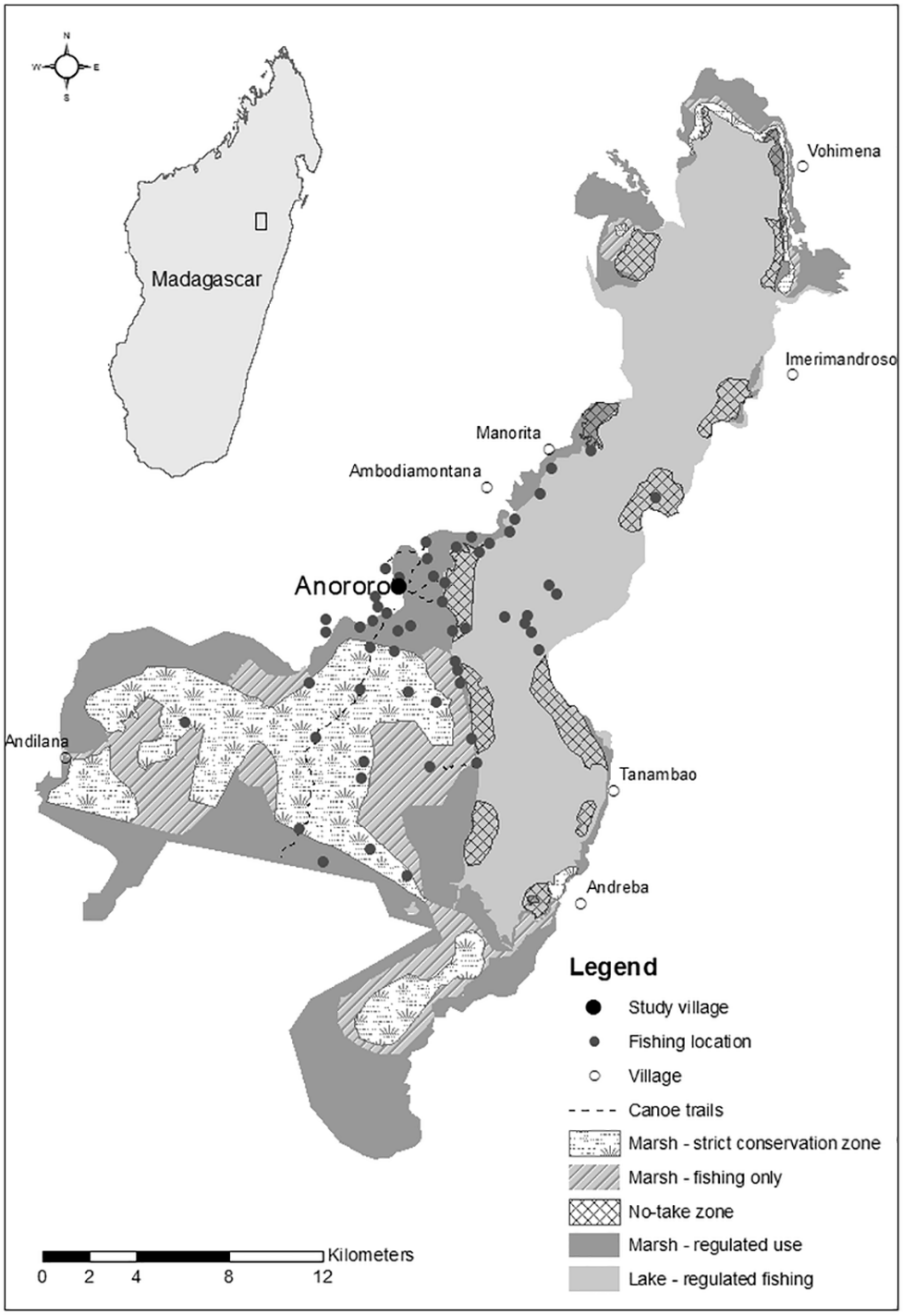
Lake Alaotra management zones. Map of Lake Alaotra showing management zones within the lake and adjacent marsh, and the centroids of fishing locations used by local fishers as recorded in the catch interview data. Restricted areas where fishing is prohibited are the strict conservation zone in the centre of the marsh and no-take zones around the lake edge.

Lake Alaotra is the largest lake in Madagascar and base for the nation’s most productive inland fishery [14]. Currently the five most predominant species, all introduced, are: Nile tilapia (*Oreochromis niloticus niloticus*), blotched snakehead (*Channa maculata*), redbreast tilapia (*Tilapia rendalli*), goldfish (*Carassius auratus auratus*), and common carp (*Cyprinus carpio carpio*) [15]. Lake Alaotra covers 200km^2^ and has a maximum depth of 4m. The marsh surrounding the lake covers 230km^2^ and approximately 1,200km^2^ of rice fields adjoin the lake and marsh (see Fig 1 [14, 16]). The entire wetland area is internationally recognised as an important area for biodiversity conservation, and was declared a Ramsar site in September 2003 and a new protected area by the government of Madagascar in 2007 [17].

This study was based in Anororo village; a community of approximately 8,000 people [18]. It was selected for its (i) large population of fishers using a variety of methods, (ii) proximity to conservation interventions, and (iii) local dependence on fishing [19]. Anororo-based fishers use a broad range of methods; the two most common are traps and gill nets, which are used in lake, lake-marsh edge, and marsh habitats. Traps are used passively overnight with usually 24 hours between fish collections. Gill nets were observed being used in three ways: (i) passively overnight, (ii) passively while waiting, and (iii) actively. Fish are sold primarily to commercial buyers from the capital city via ‘fish collectors’ who buy directly from the fishers. Local median income from fishing is approximately US$1.36 per day [15].

Current conservation interventions are based on the 2006 Lake Alaotra Management Plan, which aimed to improve the sustainability of the fishery, reduce pressures on the wetland, and conserve rare bird and mammal species, particularly the Critically Endangered Alaotran gentle lemur (see Fig 1 [20]). Fishing is prohibited in the strict conservation zone in the centre of the marsh (covering approximately 42% of the marsh) and in no-take zones around the lake edge (covering approximately 15% of the surface area of the lake). These spatial closures are additional to other regulations, including gear restrictions, minimum fish size limits, and an annual two-month fishery closure [21]. The closed period aims to protect spawning fish and allow sufficient time to prevent mouth-brooding or gravid tilapia from being harvested but is currently mis-matched with the timing of fish reproduction, largely for political reasons; the current closed period (15 November to 15 January) encompasses a key rice-farming time when employment opportunities outside fishing are high, rather than when fish spawn from late September to mid-November, when employment opportunities are low [15, 22]. We sought to understand the implications of a change in timing of the closed period prior to recommending this as a management option. The Lake Alaotra Management Plan is largely viewed as a ‘paper park’ that is failing to meet conservation goals. There is a general lack of enforcement and poor compliance with all regulations, which were drawn up with limited consideration or knowledge of likely costs to fishers [14, 19]; for example 64% of fishers interviewed fish within restricted areas, while 72% stated they fish during the closed season [15].

### Data collection

Data were collected in June and July 2009 and for 14 months from October 2009 to December 2010, comprising two dry seasons (2009 and 2010) and one wet season (2010). We conducted structured catch interviews (n = 1,800), including measurement of fish lengths (n = 27,064), opportunistically with Anororo-based fishers returning from their fishing trip and whom were willing to participate. Catch interview data were not collected during December in either year in order to retain trust between the research team and fishing community, because the current annual fishery closure was from 15 November to 15 January. A total of 537 individual fishers participated in catch interviews (approximately 70% of the estimated total number of Anororo-based fishers). We assigned respondent codes to fishers to preserve their anonymity [23]. Catch interviews were 5 to 10 minutes in duration, comprising (a) questions to determine the fisher’s fishing location, gear used, and effort that day and (b) counting and measuring fish caught.

During most catch interviews (76%), the length of each fish in the catch was measured. When catches were large (i.e., in excess of approximately 50 fish) a random sub-sample of the total catch was measured to minimise time demands on fishers (see [24]). Sub-samples of catches were then used to extrapolate out to the total catch. A sample of 498 fish obtained from fishers and fish collectors were measured and weighed separately to calculate species-specific length-weight relationships [15], which were used to estimate the total weight of a fisher’s catch.

Ethics approval for the study was granted by Imperial College Research Ethics Committee (ICREC_9_1_2). All data were collected in accordance with institutional ethics requirements, established ethical guidelines for socio-economic research, and with the consent and support of village leaders and participating fishers. Madagascar’s Department of Environment & Forest (DREF) granted permission for the research.

### Data analysis

#### Data preparation

We analysed data for 1,284 fishing trips by 284 trap fishers and 319 trips by 158 gill net fishers. To account for potential effects of different years and months on total catch weight, months were grouped in terms of water level, rainfall and season, also considering rice cultivation activities that affect level of effort put into fishing (S1 Table). The grouped months were then combined with year to achieve a single ‘Time Period’ categorical variable with eight levels (1 –May-Jun ‘09, 2 –Jul-Sep ‘09, 3 –Oct-Nov ‘09, 4 –Jan-Feb ‘10, 5 –Mar-Apr ‘10, 6 –May-Jun ‘10, 7 –Jul-Sep ‘10, 8 –Oct-Nov ‘10), which was used as a proxy to account for intra- and inter-annual changes in water level and resultant productivity, fish growth, and biomass density (see [25, 26]).

#### Analysis of catch weight

We used linear mixed effects models (LMMs) to identify factors influencing catch weight (the response variable) for fishers using traps or gill nets, including time period, fishing effort, gear type, and spatial variability. To account for differences among fishers, individual fishers (Fisher ID) were included as a random effect in both models and include repeated measurements over time for each fisher [27, 28]. While econometric production models have been criticized due to concerns about endogeneity bias [29], such production functions are widely used to represent the relationship between the dependent variable and multiple explanatory variables [30].

Nile tilapia and blotched snakehead are the predominant species in catches, accounting for 86% of fish caught and 87% of catch weight. Whole or part catches are purchased from fishers by fish collectors who estimate the value of the catch by eye depending on the size and quantity of fish (in practice, fullness of a 15L bucket as a proxy for weight in absence of weigh-scales). Because catches typically comprised a mix of species, prices were primarily determined by fish size (weight) rather than species [15]. We assessed catch as total weight for the fishing trip, reflecting economic value for the fisher (the vast majority of the catch in Lake Alaotra is sold) and the common perception that fishers are profit maximisers [31].

Traps and gill nets are the predominant methods used in Anororo and were reported during 91% of catch interviews. We analysed each of these two gear types separately and used economically relevant variables (i.e., travel time to fishing location, time spent fishing, and number of gear items used) rather than biological variables (i.e., time fishing gear is in the water) to define fisher effort. We used Pearson’s correlations as well as variance inflation factors (VIFs) to test for collinearity among fisher effort variables and gear characteristics and determine which variables should be used before starting analyses [28]. Including the total length of a trip led to large VIFs. By excluding total trip time, all variables had VIFs less than 2 indicating that very little variance inflation remained.

The response variable for both the trap and gill net models was catch weight–total weight of catch from the fishing trip. The explanatory variables (fixed effects) in each model included time period, travel time to fishing location, time spent fishing, number of gear items used, their size and mesh size, and the habitat and restricted status of the fishing location (S2 Table). Continuous variables were normalised using log transformations. Although it was not possible to include spatial structure in the models explicitly, spatial locations in the study area are closely associated with location characteristics. By including travel time to a fishing location, whether the fishing location is inside or outside of an area with restricted status in the management plan, and habitat, the models are spatially implicit.

#### Statistical inference

Information-theoretic approaches, such as AIC (Akaike’s Information Criterion), use measures of predictive power to rank models and quantify the magnitude of difference between models [32]. Rather than selecting the ‘best’ model, information-theoretic tools are used to select and average candidate or well-fitting models [33]. This approach is also robust to the mild to moderate degree of collinearity found in this study [34].

We used AIC model selection and model averaging to determine the relative importance and averaged estimates for each variable. Analyses were performed in R version 2.13.1 [35]. Global models (fitted by maximum likelihood) were run using the lme4 package in R; the MuMIn package was used for model comparison and model averaging. Following Burnham and Anderson’s [32] rule of thumb, all models where AIC differences were <4 (traps) or <6 (gill nets) were included in the candidate set of models for model averaging. AIC differences of <4 and <6 were chosen because the weight or support for subsequent models decreased considerably at this point in the trap and gill net model selection tables, respectively. No single model was clearly superior to others in the candidate set of models for either gear type, suggesting that model averaging would provide a more robust understanding of the system and reduce model selection bias effects [32].

### Estimating the impacts of spatial and temporal restrictions

Whenever possible (n = 258 occasions), fishers and collectors advised the prices received or paid, respectively, for full or partial catches. These prices were recorded on catch interview sheets. We used median fish prices and catch data for fishers using different gear types inside and outside restricted areas, as well as in the proposed closed season of October-November, to estimate the maximum short-term costs that could be incurred by fishers due to spatial and/or temporal restrictions, based on two simplifying conditions. First, we did not account for potential adaptive changes in fisher behaviour in response to the interventions or the possible overcrowding or interference that may result from such changes; and second, we did not account for potential changes in fish biomass resulting from the interventions. We focused instead on immediate short-term impacts for fishers. Although fishers stated they would adapt to spatial closures by fishing in other non-restricted locations, fishers also stated that their catches would decrease until they became familiar with the new location and because of increased fisher density. The temporal closure raises major problems for adaptation, in that the only viable alternative is to seek alternative employment during the closed period, which may not always be possible. While fishers are likely to adapt to reduce impacts, with varied and uncertain efficacy, estimating the longer-term impacts of the interventions on fish stocks would require detailed modelling of social-ecological system dynamics.

## Results

Fishers using traps fished exclusively in marsh (61%) or edge (39%) habitat, while gill net fishers primarily fished in open water at the edge (53%), in the lake (28%), and in some open areas in the marsh (19%). All fishers made only one fishing trip per day. Catch weight varied between habitats and gear types, between areas with different restricted status, and on a temporal basis, as well as between individuals; for example, mean catch weight for trap fishers was higher in edge habitat (3.37kg per trip) compared to marsh (1.84kg per trip), whereas for gill net fishers, mean catch weight was highest in lake habitat (4.13kg per trip) followed by edge (2.58kg per trip) and marsh habitat (1.17kg per trip). Catch weights in relation to fisher effort, measured as number of gear items used, time spent fishing, and travel time to fishing location, indicated that catch weight generally increases with fisher effort (S1 Fig).

### Factors affecting catch weight

**Fig 2:**
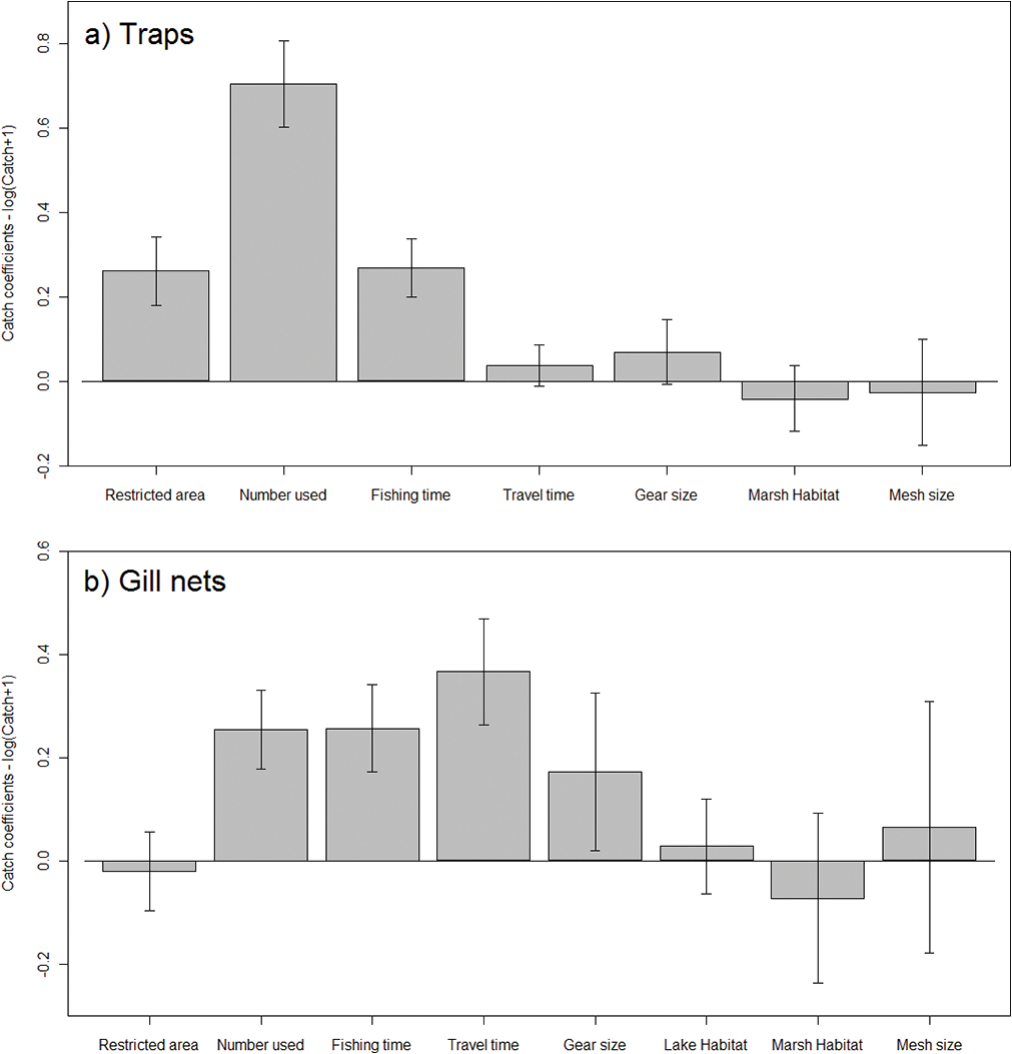
Factors affecting catch weight. Coefficient averages (± 1SE) from the candidate set of models for a) traps and b) gill nets, explaining the variation in catch weight as influenced by restricted area status (restricted versus non-restricted), fisher effort, habitat, and gear characteristics. Restricted area = fishing location is in an area with restricted status; Number used = number of gear items used; Fishing time = time spent fishing; Travel time = travel time to fishing location; Gear size = size of gear used; Lake Habitat = fishing location is in lake habitat; Marsh Habitat = fishing location is in marsh habitat; Mesh size = size of mesh of gear items used. Baseline (i.e., zero line) levels for restricted and habitat variables are ‘non-restricted’ and ‘edge habitat’, respectively. See S2 Table for variable descriptions and S5 Table for coefficient values or see http://dx.doi.org/10.6084/m9.figshare.1309446.

The model for trap fishers with the lowest AIC and most support (*W*_*i*_ = 0.14) indicated that the combined effects of the restricted area status of the fishing location, time period, and various measures of fisher effort influenced catch weight (S3 Table). Model averaging indicated that the four most important variables were restricted area status, time period, number of gear items used, and time spent fishing (Fig 2 *A*, S3 Table). Gear size and travel time to fishing location had comparatively lower variable weights. For gill net fishers, both the best model and model averaging indicated that the combined effects of time period and fisher effort (number of gear items used, travel time to fishing location, and time spent fishing) had the greatest influence on catch weight (Fig 2 *B*); gear size was less important in the models (S4 Table).

**Fig 3:**
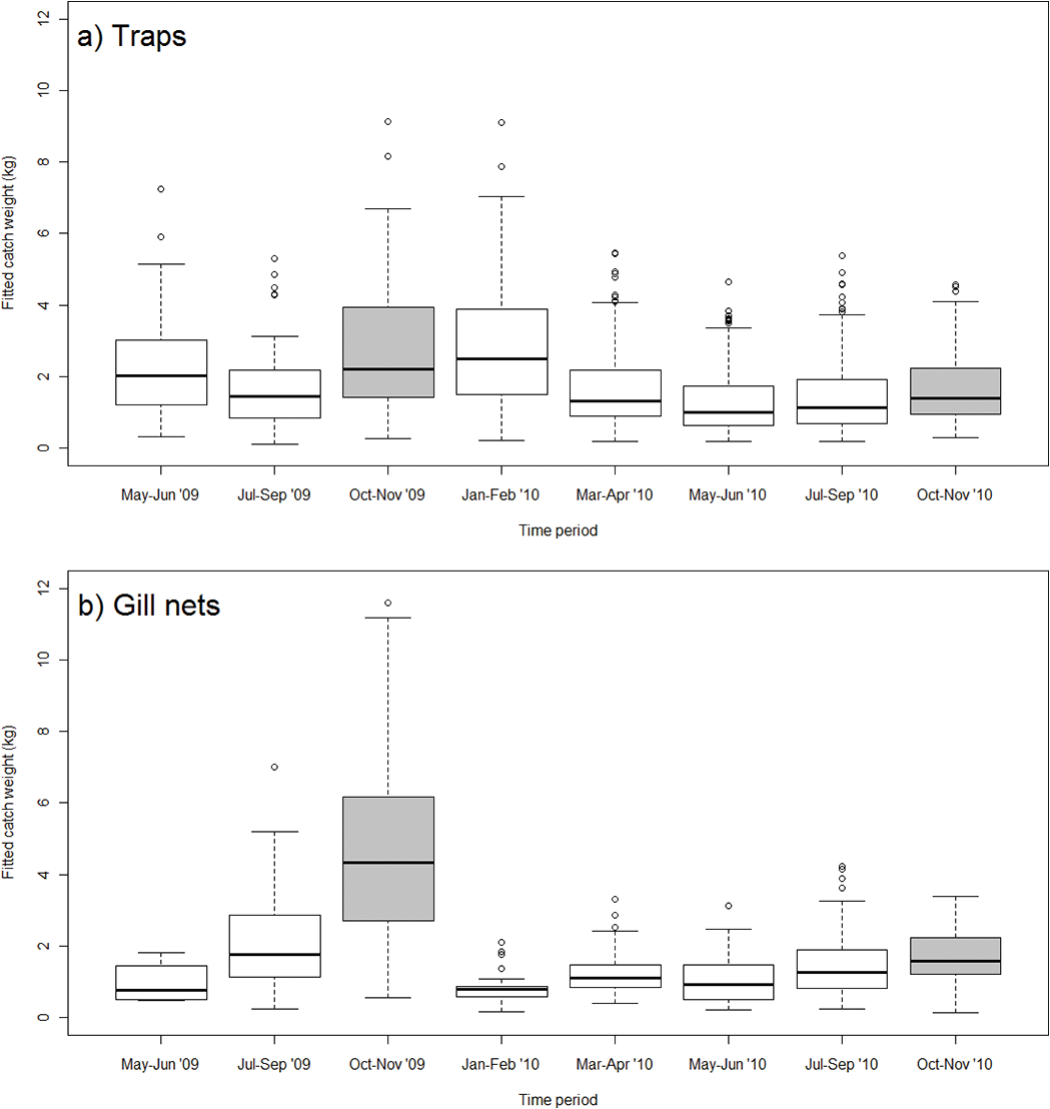
Temporal variation in catch weight. Fitted catch weight for the top model based on lowest AIC for each gear type. Box and whisker plot of modelled catch weight per trip in kilograms for a) trap fishers and b) gill net fishers over the study period; the horizontal bar represents the 50th percentile, the top of the box the 75th percentile, and the base of the box the 25th percentile. Whiskers represent the range of data, and open circles are outliers. Catch weights are largest in Jan-Feb for trap fishers and in Oct-Nov for gill net fishers. Time periods with smallest catch weight are May-Jun and Jul-Sep for trap fishers and Jan-Feb for gill net fishers. Grey shading refers to the proposed earlier closed period; the current closed period is not represented in the data.

Time period was an important variable influencing catch weight in the models for both trap fishers and gill net fishers, indicating intra- and inter-annual variability in catch weight per trip (Fig 3). Gill net fishers experienced greater temporal variation in catch weight than trap fishers. Catch weights for trap fishers were largest in the Jan-Feb month group (wet season) and smallest in the May-Jun and Jul-Sep groups (dry season), representing a 45% decrease in mean catch weight from best to worst time periods (Fig 3 *A*). In contrast, catch weights for gill net fishers were largest in the Oct-Nov group (dry season), and smallest in Jan-Feb (wet season), representing a 74% decrease in mean catch weight between the best and worst time periods (Fig 3 *B*). The high catches in Oct-Nov were a function of a high catch coefficient (S5 Table) and increased time spent fishing in those months. Focus group sessions and interviews with fishers confirmed fish are easier to catch with gill nets during the dry season because water levels are lower. Accordingly, gill net fishers spend more time fishing at that time of year to maximise catch weight and income.

### Impacts of spatial and temporal restrictions by gear type

The raw data show that trap fishers fishing in restricted areas had a larger mean catch weight than those in non-restricted areas (Table 1). Mean catch weight was largest during October-November compared to the rest of the year as well as the study period as a whole for both gear types, particularly for gill net fishers (Table 1; also see Fig 3). This suggests that spatial restrictions have greater impact on trap fishers and temporal restrictions have greater impact on gill net fishers.

**Table 1:** Impacts of spatial and temporal restrictions by gear type.

	Current	Spatial closure		Temporal closure		Both
Gear type	overall	Restricted	Non-restricted	Oct-Nov	Rest of year	closures
***Traps***						
Mean catch weight (kg) per trip (CI)	1.41 (1.32–1.51)	1.84 (1.69–2.01)	1.14 (1.04–1.24)	1.71 (1.46–1.99)	1.36 (1.26–1.46)	1.11 (1.00–1.22)
Number of trips	1,284	576	708	215	1,069	604
Total weight of all catches (kg)	3,117	1,723	1,394	632	2,485	1,149
Income for mean catch per trip^a^	US$1.18	US$1.53	US$0.95	US$1.43	US$1.13	US$0.93
***Gill nets***						
Mean catch weight (kg) per trip (CI)	1.53 (1.33–1.76)	1.40 (1.12–1.76)	1.64 (1.37–1.96)	2.62 (2.08–3.30)	1.14 (0.97–1.34)	1.21 (0.97–1.51)
Number of trips	319	137	182	114	205	118
Total weight of all catches (kg)	881	354	527	495	386	242
Income for mean catch per trip^b^	US$1.39	US$1.28	US$1.49	US$2.39	US$1.04	US$1.10
***Both gear types***						
Total trips	1,603	713	890	329	1,274	722
Total weight	3,998	2,077	1,921	1,127	2,871	1,391

Mean catch weight per trip for all catches and under spatial and temporal restrictions for each gear type. 'Current overall' shows the mean catch averaged over all locations and times of year. 'Spatial closure' compares catches in restricted areas with catches in non-restricted fishing locations. 'Temporal closure' compares catches during an October-November closed period with catches over the remainder of the year. Mean catches in non-restricted areas and not in the closed period comprise ‘both closures’. Catch weight is in kilograms (kg) with lower and upper values given for the 95% confidence interval (CI).

^a^ Median price paid for catches by trap fishers was US$0.83 per kilogram [15].

^b^ Median price paid for catches by gill net fishers was US$0.91 per kilogram [15].

If spatial restrictions were enforced, and in absence of adaptive behaviour by fishers or other changes, compliance could result in a 38% (SE±6%) decrease in mean catch weight from 1.84kg per trip (in restricted areas) to 1.14kg per trip (in non-restricted areas) for trap fishers. In contrast, mean catch weight for gill net fishers was 17% (SE±4%) larger in non-restricted areas (1.64kg per trip) than restricted areas (1.40kg per trip). Catch weights for both gear types would decrease if a closed period was enforced during October-November. However, the costs of compliance with a temporal closure differed between gear types; mean catch weight for trap fishers could decrease by 4% (SE±1%) from 1.41kg to 1.36kg per trip, whereas gill net fishers could experience a 25% (SE±7%) decrease from 1.53kg to 1.14kg per trip (Table 1). If spatial as well as temporal restrictions were strictly enforced, compliance with both would result in a 21% (SE±3%) decrease in mean catch weight (from 1.41kg to 1.11kg per trip) across trap fishers and a 21% (SE±7%) decrease (from 1.53kg to 1.21kg per trip) across gill net fishers (Table 1).

**Fig 4:**
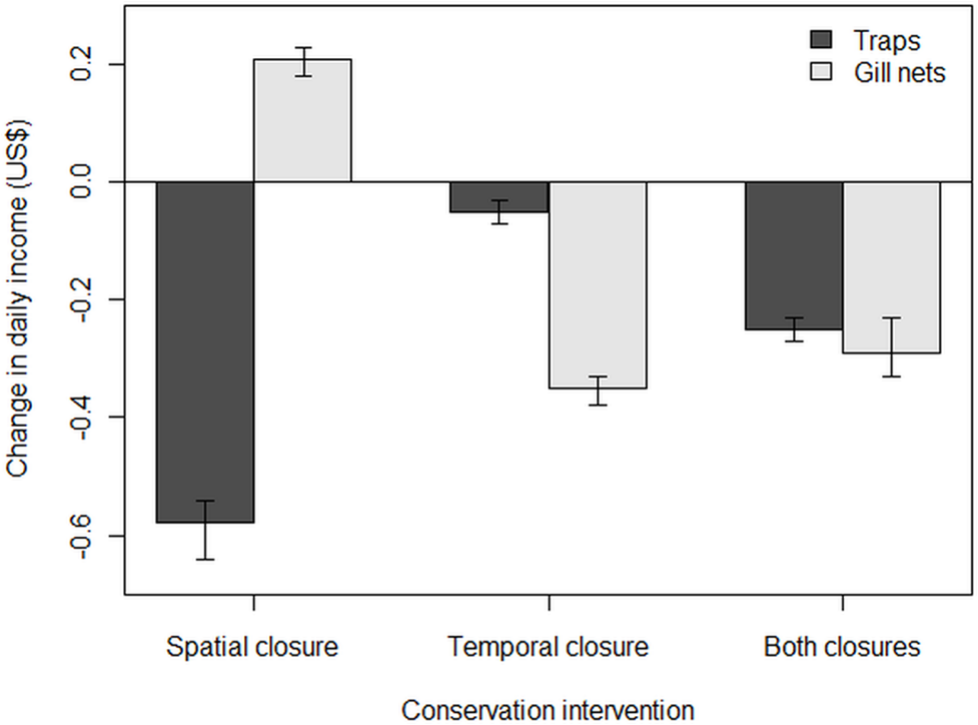
Spatial and temporal interventions and their potential costs. Potential impacts of spatial and temporal interventions, as well as both interventions combined, on the daily income of trap fishers and gill net fishers.

Based on the median price per kilogram received for catches, income loss could amount to US$0.58 per day for trap fishers and US$0.35 per day for gill net fishers (Fig 4). Compliance with both spatial and temporal closures would lead to a 55% reduction in the number of trips and a 65% reduction in catch for the fishers surveyed (Table 1).

## Discussion

Although conservation interventions often have immediate, short-term costs for local people [36], the nature of these costs and how they affect livelihoods is often unclear [37, 38]. We examined the potential impacts for local people of two conservation interventions for Lake Alaotra, spatial and temporal closures, and found that if the interventions were enforced and complied with they would not only have significant costs to fishers but also impact fisher groups differently.

Accounting for obvious predictors of catch weight (such as time spent fishing, number of gear items used, and time period), our models suggest spatial closures would have little or no adverse effect on gill net fishers but could result in smaller catch weights for trap fishers in the short-term by forcing them to fish in less favourable locations, potentially reducing their already meagre income by over one third. Conversely, a closed period in October-November would incur greater costs for gill net fishers, potentially reducing their catch and income from fishing by one quarter in the short-term because October-November appears to be the best time for fishing with gill nets.

The models confirm that greater fisher effort in terms of amount of gear used and time spent fishing results in larger catches. However, catch weight and choice of location are also influenced by gear-specific factors. The restricted areas are located in marsh and edge habitat, and trap fishers use these areas primarily because they need to affix their traps to marsh plants. Furthermore, core marsh areas where restrictions aim to protect the Alaotran gentle lemur also appear to be favourable habitat for fish [15]. In contrast, gill net fishers require open areas to avoid entanglement with vegetation, thereby travelling further onto the lake and avoiding restricted areas in the marsh and close to the lake edge. This primarily explains why trap fishers are more likely than gill net fishers to be affected by restricted areas.

Compliance with conservation interventions is critical to their effectiveness [39], but imposing interventions typically generates resentment and high levels of non-compliance [7, 40, 41], and rule-breakers can create short- and long-term costs for legitimate resource users [42]. Robust estimates of the costs of compliance can be used to improve management plans and minimise real costs to resource users [4, 43]. Within our study area, compliance with existing and previous conservation and fishery regulations has generally been very low; interviews reported in Wallace (2012) indicated that there are few alternative sources of income and food as well as considerable inertia within the fishery and a reluctance to change. Our results suggest that at least for some fishers, the scope for rapid adaptation is limited and that the costs of complying with spatial and temporal interventions may be a primary reason for non-compliance, probably in conjunction with a lack of effective enforcement (see [44]).

Our study is a baseline study that focuses on the immediate short-term costs to fishers. Consequently, we did not incorporate any measure of potential benefits from establishing restricted areas that might enhance fish abundance and catch from spill-over or indirect effects. These potential benefits are, however, unlikely to materialise in the short-term [45, 46], and the perceived immediate costs to fishers will have the greatest impact on the implementation and ongoing success of a management plan that restricts fisher behaviour [47–49]. For this reason, we did not model fisher responses to changing conditions or reserve implementation. These changes could lead to more or less severe impacts on fishers. For example, if displaced fishers enter existing fishing areas, the additional effort may reduce catches for resident fishers [8, 50]. Both spatial and temporal closures will incur immediate short-term impacts but some of these cannot be immediately adapted for. Consequently, our short-term focus is appropriate for investigating conservation interventions from fishers’ perspectives. It should also be noted that there may also be long-term costs if conservation and management interventions fail to meet their objectives.

Despite the potential long-term benefits of restricted areas and active fisheries management, the immediate and short-term costs may be too great for local people to bear and the resulting variable or weak compliance undermines conservation targets [39, 51–53]. Although economic incentives and perceptions of fairness may influence resource user behaviour [54], fishers’ motivations often reflect convenience, habit, and skill with a particular gear type and/or fishing location [15, 55]. Accordingly, differences in costs of conservation for different fisher groups suggest local management efforts need to be targeted to take the variation in such costs into account [56]. Seasonal closures are frequently less biologically effective than spatial closures [57] and compliance is lower when several regulations are implemented simultaneously [48]. Because of these findings as well as feedback from fishers, Lake Alaotra’s temporal closure is planned to be phased out in favour of spatial restrictions (R. Lewis, DWCT, pers. comm.). An alternative approach might be to increase flexibility through mobile or dynamic spatial and temporal closures, which could distribute costs and benefits more equitably among fisher groups (see [57, 58]), and be better received by fishers and ultimately more effective [6, 49]. This could include a network of smaller reserves that is responsive to shifts in fishing effort as well as factors influencing fish behaviour and production. A participatory approach incorporating input from fishers to reduce their potential costs would facilitate such a network. Data from this study may be used with spatial planning tools and scenario analysis to configure optimal reserve designs to meet various cost and biodiversity targets. This process would enhance priority-setting and adaptive management by allowing trade-offs between targets to be adjusted over time and changing conditions.

While our study estimates potential costs to fishers if spatial or temporal interventions were enforced, the full impact of restricting fishing locations may be more or less severe depending on adaptive changes in fisher behaviour and distribution [59]. Most studies focus on the ecological consequences of redistributing fishing effort [60, 61] and few consider the impacts of spatial or temporal interventions on displaced or resident fishers [62]. Redistribution of effort to non-restricted areas would increase fisher density, potentially leading to further declines in catches for displaced as well as resident fishers, at least over the short term [37, 63]. Conversely, fishers may leave the fishery, attempt to change gears, or increase effort in existing locations or at times not impacted by the intervention, potentially mitigating some effects of the intervention [59, 64].

Many millions of people depend on artisanal fishing for their livelihood throughout the year [65]. Our research provides methods to improve understanding of fishers’ spatial and temporal behaviour at a scale relevant for conservation planning. An improved understanding of fisher behaviour can inform fisheries management (see [8, 47, 66]) and contribute to conservation by ensuring that the potential impacts on resource users can be estimated and lessened during the design and planning stages of interventions. Our study provides the foundation for further analyses of fishers’ spatial behaviour, such as analysing the drivers of fisher effort, fishers’ perceptions of management interventions, and scenario analyses to understand how fishers respond and adapt to change.

## Supporting Information

S1 Fig Catch weights in relation to three measures of fisher effort. Raw catch weight (kg) for each measure of fisher effort by gear type. Catch weight generally increases with increasing fisher effort measured as number of gear items used, time spent fishing, and time spent travelling to a fishing location.
(TIF)

S1 Table ‘Time Period’ categorical variable with eight levels used as a proxy to account for intra- and inter-annual changes in water level and resultant productivity, fish growth, and biomass density. Variables used to categorise months into groups. Months marked with an asterisk are those in which data were collected in both years (2009 and 2010). Water level categories reflect the average range of values above the mean lowest water level: Highest = +1.6m to +2.0m; High = +0.9m to +1.69m; Medium = +0.2m to +0.89m; Low = 0m to +0.19m. Mean rainfall: High >150mm; Medium = 30mm to 149mm; Low = 10mm to 29mm; Very low <10mm.
(DOCX)

S2 Table Variables used in each linear mixed effects model. List, type, and description of variables used to predict catch weight in two separate LMMs for trap and gill net fishers.
(DOCX)

S3 Table Coefficients for factors affecting catch weight for fishers using traps in the 16 top models used in model averaging. Coefficients for the fixed effects of the 16 most parsimonious models that were used in model averaging to identify factors influencing catch weight for fishers using traps. A ‘+’ indicates that a factor variable was included in the model, whereas a blank field means that the variable was not included. Coefficients cannot be presented for factor variables (see S5 Table). The number of parameters in the model (k), the AIC and AIC difference (ΔAIC), and weight (*W*_*i*_) is given for each model. Individual variable weights (*w*_*i*_) are also provided.
(DOCX)

S4 Table Coefficients for factors affecting catch weight for fishers using gill nets in the 14 top models used in model averaging. Coefficients for the fixed effects of the 14 most parsimonious models that were used in model averaging to identify factors influencing catch weight for fishers using gill nets. A ‘+’ indicates that a factor variable was included in the model, whereas a blank field means that the variable was not included. Coefficients cannot be presented for factor variables (see S5 Table). The number of parameters in the model (k), the AIC and AIC difference (ΔAIC), and weight (*W*_*i*_) is given for each model. Individual variable weights (*w*_*i*_) are also provided.
(DOCX)

S5 Table Averaged model results for each gear type. Averaged model parameters explaining catch weight for trap and gill net fishers. The coefficients, standard error, and lower and upper confidence intervals for each variable are provided for each averaged set of models. Baseline levels for restricted, time period, and habitat variables for both models are ‘non-restricted’, ‘Time period May-Jun ‘09’, and ‘edge’, respectively.
(DOCX)
