# Supplementary figures and images for: Quantifying the Short-Term Costs of Conservation Interventions for Fishers at Lake Alaotra, Madagascar
Source: PLoS One. 2015 Jun 24;10(6):e0129440. doi: 10.1371/journal.pone.0129440 (PMC4481106; doi:10.1371/journal.pone.0129440)

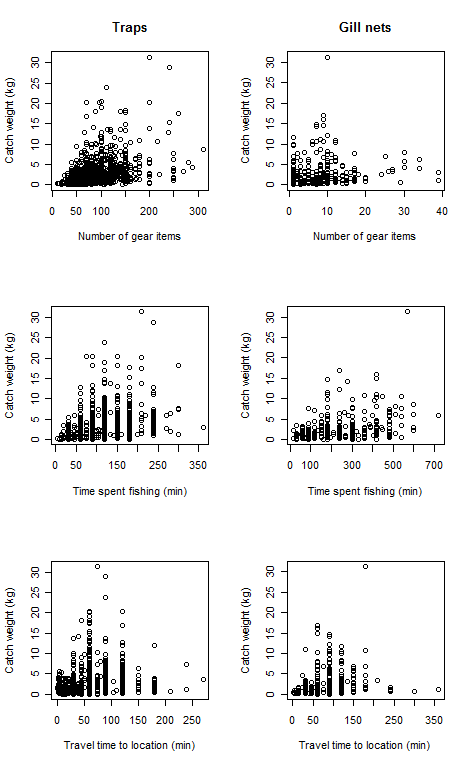

Supplement: S1 Fig — Raw catch weight (kg) for each measure of fisher effort by gear type. Catch weight generally increases with increasing fisher effort measured as number of gear items used, time spent fishing, and time spent travelling to a fishing location. (TIF) [file pone.0129440.s001.tif]
