# Supplementary material for: Quantifying the Short-Term Costs of Conservation Interventions for Fishers at Lake Alaotra, Madagascar
Source: PLoS One. 2015 Jun 24;10(6):e0129440. doi: 10.1371/journal.pone.0129440 (PMC4481106; doi:10.1371/journal.pone.0129440)
Supplement: S1 Table — Variables used to categorise months into groups. Months marked with an asterisk are those in which data were collected in both years (2009 and 2010). Water level categories reflect the average range of values above the mean lowest water level: Highest = +1.6m to +2.0m; High = +0.9m to +1.69m; Medium = +0.2m to +0.89m; Low = 0m to +0.19m. Mean rainfall: High >150mm; Medium = 30mm to 149mm; Low = 10mm to 29mm; Very low <10mm. (DOCX) [file pone.0129440.s002.docx]

**S1 Table. ‘Time Period’ categorical variable with eight levels used as a proxy to account for intra- and inter-annual changes in water level and resultant productivity, fish growth, and biomass density.** Variables used to categorise months into groups. Months marked with an asterisk are those in which data were collected in both years (2009 and 2010). Water level categories reflect the average range of values above the mean lowest water level: Highest = +1.6m to +2.0m; High = +0.9m to +1.69m; Medium = +0.2m to +0.89m; Low = 0m to +0.19m. Mean rainfall: High >150mm; Medium = 30mm to 149mm; Low = 10mm to 29mm; Very low <10mm.

| **Group** | **Month** | **Water level^a^** | **Rainfall^b^** | **Season** | **Rice cultivation activities^c^** |
| --- | --- | --- | --- | --- | --- |
| Jan-Feb | January | High – increasing | High | Wet | 1^st^ season planting |
|  | February | High – increasing | High | Wet | 1^st^ season planting |
| Mar-Apr | March | Highest | Medium | Wet | 1^st^ season maintenance |
|  | April | Highest | Medium | Wet | 1^st^ season maintenance |
| May-Jun | May | High – decreasing | Low | Dry | 1^st^ season harvesting |
|  | June* | High – decreasing | Very low | Dry | 1^st^ season harvesting |
| Jul-Sep | July* | Medium – decreasing | Very low | Dry | 2^nd^ season planting |
|  | August | Medium – decreasing | Very low | Dry | 2^nd^ season planting |
|  | September | Medium – decreasing | Very low | Dry | 2^nd^ season planting |
| Oct-Nov | October* | Low – still decreasing | Medium | Dry | 2^nd^ season maintenance; 1^st^ season prep. |
|  | November* | Lowest | Medium | Dry | 2^nd^ season maintenance; 1^st^ season prep. |
| n/a | December^d^ | Medium – increasing | High | Wet | 2^nd^ season harvest; 1^st^ season prep. |

^a^ Source: this study and Ferry ([2009](#_ENREF_1)).

^b^ Source: TAMSAT rainfall data ([Grimes et al. 1999](#_ENREF_2)).

^c^ Source: this study and Le Courtois ([2010](#_ENREF_3)).

^d^ Catch interviews were not conducted during the month of December. If data were available, and based on the above criteria, December would be grouped with Jan-Feb.

n/a = not applicable.

**References**

Ferry, L., Mietton, M., Robison, L., and Erismann, J. (2009) Le lac Alaotra à Madagascar - Passé, présent et futur. *Zeitschrift für Geomorphologie* **53**:299-318.

Grimes, D.I.F., Pardo-Igúzquiza, E., and Bonifacio, R. (1999) Optimal areal rainfall estimation using rainguages and satellite data. *Journal of Hydrology* **222**:93-108.

Le Courtois, S. (2010) Household choices in rice cultivation in a social-ecological system and impacts on productivity: Lessons from Anororo, Lac Alaotra, Madagascar. Master of Science Thesis. Imperial College London, London. 79 pp.
