# Supplementary material for: Quantifying the Short-Term Costs of Conservation Interventions for Fishers at Lake Alaotra, Madagascar
Source: PLoS One. 2015 Jun 24;10(6):e0129440. doi: 10.1371/journal.pone.0129440 (PMC4481106; doi:10.1371/journal.pone.0129440)
Supplement: S2 Table — List, type, and description of variables used to predict catch weight in two separate LMMs for trap and gill net fishers. (DOCX) [file pone.0129440.s003.docx]

**S2 Table. Variables used in each linear mixed effects model.** List, type, and description of variables used to predict catch weight in two separate LMMs for trap and gill net fishers.

| **Variable** | **Type** | **Description** |
| --- | --- | --- |
| ***Response variable*** | | |
| Catch | Continuous | Grams of fish caught during a fishing trip at a given location (location is as defined by fishers). |
| ***Explanatory variables (fixed effects)*** | | |
| Time period | Categorical | 8-level factor; combines year (2009 or 2010) and months grouped according to water level, rainfall, season, and timing of rice cultivation activities. |
| Travel time | Continuous | Estimated travel time in minutes from the village to the fishing location. |
| Fishing time | Continuous | Estimated time in minutes spent fishing (removing fish from traps or gill nets after arriving at the fishing location and before beginning to travel back to the village). |
| Number used | Continuous | Estimated number of gear items used on the trip. |
| Gear size | Continuous | Size of gear item: average volume of a trap (m^3^) or average area of a gill net (m^2^). |
| Mesh size | Continuous | Mesh size in millimetres. |
| Habitat | Categorical | 2-level factor (traps: marsh and lake-marsh edge) or 3-level factor (gill nets: marsh, lake-marsh edge, and lake); traps are not used in lake habitat. Fishing location used on a trip is within a single habitat. |
| Restricted | Categorical | 2-level factor; restricted area status: fishing location is inside (1) or outside (0) of the strict conservation zone in the marsh or the no-take zones (NTZs) at the lake edge. |
| ***Random effect*** | | |
| FisherID | Categorical | Individual fishers were identified by a unique ID and may have multiple fishing trips within the datasets. |
