# Supplementary material for: Quantifying the Short-Term Costs of Conservation Interventions for Fishers at Lake Alaotra, Madagascar
Source: PLoS One. 2015 Jun 24;10(6):e0129440. doi: 10.1371/journal.pone.0129440 (PMC4481106; doi:10.1371/journal.pone.0129440)
Supplement: S4 Table — Coefficients for the fixed effects of the 14 most parsimonious models that were used in model averaging to identify factors influencing catch weight for fishers using gill nets. A ‘+’ indicates that a factor variable was included in the model, whereas a blank field means that the variable was not included. Coefficients cannot be presented for factor variables (see S5 Table). The number of parameters in the model (k), the AIC and AIC difference (ΔAIC), and weight (W i) is given for each model. Individual variable weights (w i) are also provided. (DOCX) [file pone.0129440.s005.docx]

**S4 Table. Coefficients for factors affecting catch weight for fishers using gill nets in the 14 top models used in model averaging.** Coefficients for the fixed effects of the 14 most parsimonious models that were used in model averaging to identify factors influencing catch weight for fishers using gill nets. A ‘+’ indicates that a factor variable was included in the model, whereas a blank field means that the variable was not included. Coefficients cannot be presented for factor variables (see S5 Table in the Supporting Information for averaged model parameters). The number of parameters in the model (k), the AIC and AIC difference (ΔAIC), and weight (*W_i_*) is given for each model. Individual variable weights (*w_i_*) are also provided.

| Intercept | Restricted | Time period | Number used | Fishing time | Travel time | Gear size | Habitat | Mesh size | k | AIC | ΔAIC | Model weights (*W_i_*) |
| --- | --- | --- | --- | --- | --- | --- | --- | --- | --- | --- | --- | --- |
| 2.835 |  | + | 0.2469 | 0.2453 | 0.3787 | 0.2557 |  |  | 14 | 945.9 | 0.00 | 0.26 |
| 2.858 | + | + | 0.2471 | 0.2417 | 0.3810 | 0.2527 |  |  | 15 | 947.6 | 1.95 | 0.10 |
| 2.353 |  | + | 0.2395 | 0.2450 | 0.3700 | 0.2403 |  | 0.1643 | 15 | 947.7 | 2.05 | 0.09 |
| 3.272 |  | + | 0.2379 | 0.2576 | 0.3066 | 0.2328 | + |  | 16 | 947.6 | 2.15 | 0.09 |
| 3.569 |  | + | 0.2936 | 0.2795 | 0.4180 |  |  |  | 13 | 948.3 | 2.17 | 0.09 |
| 2.301 |  | + | 0.2698 | 0.2741 | 0.3911 |  |  | 0.3954 | 14 | 949.3 | 3.36 | 0.05 |
| 3.997 |  | + | 0.2789 | 0.2899 | 0.3300 |  | + |  | 15 | 949.2 | 3.47 | 0.05 |
| 2.322 | + | + | 0.2390 | 0.2411 | 0.3714 | 0.2353 |  | 0.1834 | 16 | 949.5 | 3.97 | 0.04 |
| 3.350 | + | + | 0.2379 | 0.2570 | 0.3043 | 0.2301 | + |  | 17 | 949.2 | 3.99 | 0.04 |
| 3.585 | + | + | 0.2937 | 0.2754 | 0.4198 |  |  |  | 14 | 949.9 | 4.04 | 0.03 |
| 2.927 |  | + | 0.2329 | 0.2574 | 0.3013 | 0.2222 | + | 0.1158 | 17 | 949.6 | 4.31 | 0.03 |
| 2.941 |  | + | 0.2605 | 0.2855 | 0.3118 |  | + | 0.3241 | 16 | 950.5 | 5.03 | 0.02 |
| 2.262 | + | + | 0.2692 | 0.2693 | 0.3917 |  |  | 0.4130 | 15 | 950.8 | 5.14 | 0.02 |
| 4.074 | + | + | 0.2783 | 0.2891 | 0.3271 |  | + |  | 16 | 950.7 | 5.23 | 0.02 |
| Individual variable weights (*w_i_*) | 0.26 | 1.00 | 1.00 | 1.00 | 1.00 | 0.70 | 0.26 | 0.27 |  |  |  |  |
