# Supplementary material for: Quantifying the Short-Term Costs of Conservation Interventions for Fishers at Lake Alaotra, Madagascar
Source: PLoS One. 2015 Jun 24;10(6):e0129440. doi: 10.1371/journal.pone.0129440 (PMC4481106; doi:10.1371/journal.pone.0129440)
Supplement: S5 Table — Averaged model parameters explaining catch weight for trap and gill net fishers. The coefficients, standard error, and lower and upper confidence intervals for each variable are provided for each averaged set of models. Baseline levels for restricted, time period, and habitat variables for both models are ‘non-restricted’, ‘Time period May-Jun ‘09’, and ‘edge’, respectively. (DOCX) [file pone.0129440.s006.docx]

**S5 Table. Averaged model results for each gear type.** Averaged model parameters explaining catch weight for trap and gill net fishers. The coefficients, standard error, and lower and upper confidence intervals for each variable are provided for each averaged set of models. Baseline levels for restricted, time period, and habitat variables for both models are ‘non-restricted’, ‘Time period May-Jun ‘09’, and ‘edge’, respectively.

| **Model** | **Coefficient** | **SE** | **Lower CI** | **Upper CI** |
| --- | --- | --- | --- | --- |
| ***Traps*** |  |  |  |  |
| (Intercept) | 3.2100 | 0.6380 | 1.960 | 4.460 |
| Restricted area | 0.2610 | 0.0812 | 0.102 | 0.420 |
| Time period Jul-Sep ‘09 | -0.4050 | 0.1850 | -0.769 | -0.042 |
| Time period Oct-Nov ‘09 | 0.2460 | 0.1800 | -0.106 | 0.598 |
| Time period Jan-Feb ‘10 | 0.2010 | 0.1670 | -0.126 | 0.528 |
| Time period Mar-Apr ‘10 | -0.2110 | 0.1610 | -0.527 | 0.106 |
| Time period May-Jun ‘10 | -0.4310 | 0.1630 | -0.751 | -0.111 |
| Time period Jul-Sep ‘10 | -0.3650 | 0.1590 | -0.677 | -0.053 |
| Time period Oct-Nov ‘10 | -0.1930 | 0.1740 | -0.533 | 0.148 |
| log(Number used) | 0.7050 | 0.1030 | 0.503 | 0.906 |
| log(Fishing time) | 0.2680 | 0.0694 | 0.132 | 0.404 |
| log(Travel time) | 0.0374 | 0.0484 | -0.057 | 0.132 |
| log(Gear size) | 0.0694 | 0.0757 | -0.079 | 0.218 |
| Habitat Marsh | -0.0418 | 0.0779 | -0.195 | 0.111 |
| log(Mesh size) | -0.0263 | 0.1250 | -0.272 | 0.219 |
| ***Gill nets*** |  |  |  |  |
| (Intercept) | 2.9800 | 1.0700 | 0.874 | 5.080 |
| Restricted area | -0.0200 | 0.0762 | -0.169 | 0.129 |
| Time period Jul-Sep ‘09 | 0.3810 | 0.4450 | -0.492 | 1.250 |
| Time period Oct-Nov ‘09 | 1.0200 | 0.4420 | 0.153 | 1.880 |
| Time period Jan-Feb ‘10 | -0.8210 | 0.4680 | -1.740 | 0.096 |
| Time period Mar-Apr ‘10 | -0.2500 | 0.4470 | -1.130 | 0.627 |
| Time period May-Jun ‘10 | -0.0665 | 0.4480 | -0.944 | 0.811 |
| Time period Jul-Sep ‘10 | -0.1420 | 0.4310 | -0.987 | 0.703 |
| Time period Oct-Nov ‘10 | -0.2500 | 0.4480 | -1.130 | 0.627 |
| log(Number used) | 0.2550 | 0.0761 | 0.105 | 0.404 |
| log(Fishing time) | 0.2570 | 0.0849 | 0.091 | 0.424 |
| log(Travel time) | 0.3670 | 0.1030 | 0.165 | 0.568 |
| log(Gear size) | 0.1720 | 0.1530 | -0.128 | 0.471 |
| Habitat Lake | 0.0280 | 0.0920 | -0.152 | 0.208 |
| Habitat Marsh | -0.0727 | 0.1650 | -0.397 | 0.252 |
| log(Mesh size) | 0.0650 | 0.2440 | -0.413 | 0.543 |
